# Effects on Tissue Integration of Collagen Scaffolds Used for Local Delivery of Gentamicin in a Rat Mandible Defect Model

**DOI:** 10.3390/bioengineering9070275

**Published:** 2022-06-24

**Authors:** Caroline Billings, Austin J. Bow, Steven D. Newby, Robert L. Donnell, Madhu Dhar, David E. Anderson

**Affiliations:** College of Veterinary Medicine, University of Tennessee, Knoxville, TN 37996, USA; abow@vols.utk.edu (A.J.B.); snewby@vols.utk.edu (S.D.N.); rdonnell@utk.edu (R.L.D.); mdhar@utk.edu (M.D.); dander48@utk.edu (D.E.A.)

**Keywords:** surgical site infections, collagen matrix, local drug delivery, tissue regeneration, gentamicin, mandibular model, drug elution, biocompatibility

## Abstract

Surgical site infections (SSIs) are a common complication following orthopedic surgery. SSIs may occur secondary to traumatic or contaminated wounds or may result from invasive procedures. The development of biofilms is often associated with implanted materials used to stabilize injuries and to facilitate healing. Regardless of the source, SSIs can be challenging to treat. This has led to the development of devices that act simultaneously as local antibiotic delivery vehicles and as scaffolds for tissue regeneration. The goal for the aforementioned devices is to increase local drug concentration in order to enhance bactericidal activity while reducing the risk of systemic side effects and toxicity from the administered drug. The aims of this study were to assess the effect of antibiotic loading of a collagen matrix on the tissue integration of the matrix using a rat mandibular defect model. We hypothesized that the collagen matrix could load and elute gentamicin, that the collagen matrix would be cytocompatible in vitro, and that the local delivery of a high dose of gentamicin via loaded collagen matrix would negatively impact the tissue–scaffold interface. The results indicate that the collagen matrix could load and elute the antimicrobial gentamicin and that it was cytocompatible in vitro with or without the presence of gentamicin and found no significant impact on the tissue–scaffold interface when the device was loaded with a high dose of gentamicin.

## 1. Introduction

Surgical site infections (SSIs) are a common yet potentially devastating complication following surgery [1]. SSIs are most frequently caused by bacterial organisms, typically Gram-positive Staphylococcal bacterial species, such as *Staphylococcus aureus (S. aureus)* [2,3,4], although Gram-negative organisms such as *Pseudomonas*, *Enterococcus*, and *Escherichia coli* also cause infections [2,3]. Orthopedic surgery is accompanied by a significant risk of SSI, with an estimated 31,000–35,000 cases annually in the United States [2]. Infection risk is multifactorial. Risk is partially due to the nature of orthopedic surgery, as orthopedic procedures often require indwelling hardware to stabilize bones, repair fractures or replace joints [2]. Indwelling hardware is known to be at risk of microbial contamination and subsequent biofilm formation or chronic infection for the lifetime of the indwelling device [5]. Infection risk is also a result of the patient population and presentation. Individuals requiring joint replacements may possess comorbidities, increasing their likelihood of SSI, and presentation may involve trauma or contaminated injuries, also increasing the risk of bacterial infection [1,6].

SSIs can cause significant morbidity and mortality to the patient [3,7], particularly because treatment relies heavily upon systemic antimicrobial therapy and surgical revision procedures [8,9], which may leave patients suffering adverse side effects from systemic antibiotics [9] or with impaired function from tissue loss or implant removal during surgical revisions. Traditional treatment strategies often fall short of a cure, which leads to persistent bacterial infection. This may be due to antimicrobial-resistant bacterial species, such as methicillin-resistant *S. aureus* (MRSA) [4], or to inadequate antimicrobial penetration, whether secondary to inadequate tissue penetration [9,10] or metabolically inactive bacteria safely sequestered in biofilms [2,9], as well as to recurrent bacterial infection that can occur due to indwelling devices [11,12,13], which pose a risk of bacterial infection for the lifetime of the device. For these same reasons, SSIs also place a substantial burden on the healthcare system [3,7]. The estimated annual cost of managing SSIs is reported by the Centers of Disease Control (CDC) to be USD 3.3 billion [7], and significant SSIs are recognized to increase the duration of hospitalization by an average of 9.7 days [7].

New strategies to overcome current limitations in the treatment of SSI are needed, and locally implantable medical devices used to deliver antimicrobials may help to prevent bacterial colonization of tissues. Recently, interest has increased for the use of scaffolds that can simultaneously aid in tissue regeneration and serve as local drug delivery devices [9,14,15]. Devices that are biocompatible and bioresorbable are of particular interest in order to reduce concerns of a foreign body response and to eliminate the need for revision procedures to remove the implanted device [13,16]. An ideal device within this class is able to deliver sufficiently high concentrations of antimicrobials to the surrounding tissues to overcome the hurdle of inadequate tissue penetration and to impede biofilm formation [16,17], all while utilizing a dose of antimicrobials that will not injure the surrounding tissues or impair tissue healing [15,18,19].

Collagen is frequently used in biomedical applications and is of extreme interest for use as a tissue regeneration and drug delivery device [20,21]. Collagen is one of the most abundant proteins in the body and is a major component of the extracellular matrix (ECM) [22]. The ECM serves to organize cells in a 3D space and to provide attachment points and environmental signals for tissue development. Functionally, natural collagen within the body provides extensive mechanical support. Collagen is also recognized to be involved in many other tissue functions, including tissue repair [21]. When utilizing collagen as a biomaterial, there are many variations to choose from, and it is necessary to process the collagen to ensure that it is safe and non-immunogenic to the recipient. Additionally, the collagen will most likely need to be modified in some way, such as cross-linked with elastin, to slow degradation rates and to add elasticity to complement the stiffness provided by collagen [23]. One of the most common forms of collagen used in commercial biomaterial scaffolds is xenogenic collagen of porcine origin [24]. There are many variations of porcine collagen matrices developed for various indications, but the primary goals of collagen matrices are to provide: excellent biocompatibility, a highly porous structure to allow for tissue ingrowth and matrix incorporation, mechanical properties similar to tissues of interest, and degradation properties that match the speed of tissue regeneration [23].

Within this work, our goal was to evaluate the utility of a commercially available collagen matrix (Fibro-Gide^®^, Geistlich Pharma North America, Inc., Princeton, NJ, USA) typically used for soft-tissue regeneration as a cytocompatible, therapeutically effective drug delivery device, with a special emphasis on effects of a high dose delivery of gentamicin on the tissue–scaffold interface. We hypothesized that loading tissue regeneration scaffolds with high doses of antibiotics, known to be cytotoxic, would result in decreased tissue integration of the scaffold. Our objectives of these experiments using the chosen collagen matrix were: (1) to assess loading and elution of gentamicin from the matrix, (2) to evaluate cytocompatibility, in vitro, of the matrices in the presence of antibiotics, and (3) to determine if loading with a high dose of the antibiotic within the device would negatively impact the tissue–scaffold interface and subsequent tissue integration in vivo.

## 2. Materials and Methods

### 2.1. Material Description

Commercially available porcine collagen matrix (Geistlich Fibro-Gide^®^, Geistlich Pharma AG, Wolhusen, Switzerland) was utilized for in vitro and in vivo experiments. This material is described by the manufacturer as a porous, resorbable, volume-stable matrix composed of reconstituted, chemically cross-linked collagen that is intended for soft tissue augmentation procedures [25]. Fibro-Gide^®^ has one porous layer that consists of 60–96% (*w/w*) porcine collagen (types I and III) and 4–40% (*w/w*) elastin. Average pore diameter is 92 μm, and the material possesses 93% volume porosity with interconnected pores [24]. Fibro-Gide is provided as a sterilized block that varies in length and width but has a fixed height of 6 mm. For the purpose of these experiments, Fibro-Gide^®^ (15 mm × 20 mm × 6 mm) was handled in sterile conditions and sectioned into 5 mm diameter × 6 mm height cylinders (V = π r^2^ h = π·2.5^2^·6 ≈ 117.8) utilizing a sterile 5 mm biopsy punch. Scanning electron microscopy (SEM) images of Fibro-Gide^®^ cylinders were obtained utilizing a Zeiss EVO MA15 scanning electron microscope (UT Institute for Advanced Material Manufacturing).

### 2.2. Antimicrobial Selection

Gentamicin sulfate solution (100 mg/mL) was utilized for these experiments. Gentamicin is an aminoglycoside antibiotic with bactericidal activity against a range of Gram-negative bacterial species and some methicillin-susceptible *S. aureus* species [26]. This antibiotic was largely chosen due to the use of gentamicin to prevent and treat surgical site infections, whether administration is systemic or accomplished via a local drug delivery device [17,27,28,29,30]. Gentamicin was also selected for the well-characterized toxicity profile in rats [31,32], possession of cytotoxic behavior at high concentrations in vitro [15], as well as the highly concentrated drug formulation, which facilitated higher loading doses onto the Fibro-Gide^®^ collagen matrix.

### 2.3. In Vitro Drug Loading and Elution

#### 2.3.1. Hydrophilicity

Hydrophilic properties of the material were determined by calculating the percent equilibrium water content (EWC) (Equation (1)). Phosphate-buffered saline (PBS) was added in 10 μL increments until the devices were saturated and wet weights were recorded.

(1) $$EWC\;\left(\%\right)=\frac{\left(Weight\;hydrated\;sample-weight\;dry\;sample\right)}{Weight\;hydrated\;sample}\times 100$$

Based on the determined hydrophilic properties, the loading volume of gentamicin (100 mg/mL) was calculated. The optimal loading volume calculated, which utilized 100 μL/5 mm diameter and 6 mm height cylinder.

#### 2.3.2. Drug Loading

Experimental Fibro-Gide^®^ cylinders were loaded with either a high (10 mg) or low (5 mg) dose of gentamicin, (n = 3 cylinders/dose). To load 10 mg of gentamicin, 100 μL of 100 mg/mL gentamicin solution was applied to experimental cylinders (n = 3) under sterile conditions. To ensure an equal volume across each cylinder, gentamicin (100 mg/mL) was diluted 1:1 with sterile water for injection to create a 50 mg/mL solution. Then, 100 μL of the resulting 50 mg/mL solution was applied to the experimental cylinders (n = 3) to load 5 mg of gentamicin. Once loaded with gentamicin, cylinders were incubated at room temperature for four hours.

#### 2.3.3. Drug Elution Protocol

Once loaded, cylinders were completely submerged in PBS (2 mL) and incubated in a water-jacketed incubator at 37 °C to mimic physiologic temperature. Using protocols established in our laboratory, supernatant was collected via complete media removal at pre-determined timepoints (3, 24, and 48 h and on days: 3, 4, 6, 8, 10, 12 and 14). At each timepoint, 2 mL of fresh PBS was replaced, and devices were returned to incubation.

#### 2.3.4. Drug Concentration

The concentration of gentamicin in eluent samples from gentamicin-impregnated Fibro-Gide^®^ was determined using ultra high-pressure liquid chromatography (UHPLC) with mass spectrometry detection after dilution of the PBS samples with an internal standard solution (Analytical Chemistry Services, College of Veterinary Medicine, Service, Iowa State University, Ames, IA, USA). The UHPLC consisted of an UltiMate 3000 Pump, Column Compartment and Autosampler (Thermo Scientific, San Jose, CA, USA) coupled to an Orbitrap mass spectrometer (Q Exactive Focus, Thermo Scientific, San Jose, CA, USA). The analysis was performed by hydrophilic interaction chromatography (HILIC) with a ZIC HILIC column, 150 mm × 2.1 mm, 5 µm particles (Merck KGaA, Darmstadt, Germany through EMD Millipore, MA, USA). Gentamicin consists of a mixture of four components: Gentamicin C1, 0.767 fraction of total; gentamicin C2/2a, 0.175 fraction; gentamicin C1a, 0.058 fraction. Calibration curves for gentamicin C1 and gentamicin C2/2a exhibited a correlation coefficient (r2) exceeding 0.995 across the concentration range. One of three calibration curves for gentamicin C1a had a correlation coefficient (r2) in the 0.985 range, while the others exhibited r2 exceeding 0.991. The limit of quantitation (LOQ) was 0.04 µg/mL for gentamicin C1 and 0.01 µg/mL for the other two gentamicin components. The limit of detection (LOD) was 0.01 µg/mL for gentamicin C1 and 0.005 µg/mL for the other two gentamicin components.

### 2.4. In Vitro Cytocompatibility

#### 2.4.1. Cell Culture Conditions

Commercially obtained MC3T3-E1 cells (ATCC) were utilized for all in vitro assays, as previously described by Jackson et al. [33] and Bow et al. [34]. Cells were expanded in tissue culture polystyrene flasks at 37 °C and 5% CO_2_ in αMEM media with 10% fetal bovine serum (FBS) and 1% penicillin streptomycin (pen–strep). Media were changed every 2 to 3 days. Once cell cultures had reached approximately 90% confluency, cells underwent enzymatic release from the growth substrate utilizing 0.25% Trypsin–EDTA solution for 2 min at 37 °C. Cells were collected and allocated to experimental set-up.

#### 2.4.2. Cell Seeding to Scaffolds

Fibro-Gide^®^ cylinders were sharply sectioned into wafers (5 mm× 1.5 mm) under sterile conditions. Each wafer was then placed into an individual well of a non-treated polystyrene plate and seeded individually with 5.0 × 10^4^ of MC3T3-E1 cells/15 μL of growth media. Seeded wafers were allowed to incubate at room temperature for 30 min to allow for cells to infiltrate the wafer. After 30 min, 0.5 mL of one of three variations of cell culture media was added. Media variations are as follows: (1) αMEM with 10% FBS and 1% pen-strep, (2) αMEM with 10% FBS and 20 μg/mL of gentamicin, and (3) αMEM with 10% FBS and 200 μg/mL of gentamicin. After media addition, plates were incubated at 37 °C and 5% CO_2_. Control parameters were provided by plating 1.0 × 10^4^ MC3T3-E1 cells without Fibro-Gide^®^ wafers in tissue culture-treated plates with each of the three described media variations (positive control). Negative controls were provided by Fibro-Gide^®^ wafers and tissue culture-treated plates without any cells seeded. Plates were incubated for 3, 5 and 7 days to facilitate in vitro assays for cellular adhesion and proliferation.

#### 2.4.3. Cell Adhesion and Proliferation

Calcein-AM staining was performed to determine cellular adhesion and viability on Fibro-Gide^®^ wafers. At 3, 5 and 7 days, samples (n = 3/variation/timepoint) were incubated with 2 μg/mL calcein-AM staining solution at 37 °C for five minutes. Fluorescent images of all samples at each time point were taken to verify the presence and viability of cells [33]. The vast majority of cells attached to the underside of the Fibro-Gide^®^ wafers and were visualized after the wafers were gently turned over (180°) utilizing sterile forceps.

MTS assay (3-(4,5-dimethylthiazol-2-yl)-5-(3-carboxymethoxyphenyl)-2-(4-sulfophenyl)-2*H*-tetrazolium) [34] was performed to determine cell proliferation on Fibro-Gide^®^ wafers. At 3, 5 and 7 days, 100 μL of MTS reagent was added to samples (n = 3/variation/timepoint) within 0.5 mL indwelling media. Samples were incubated for three hours at 37 °C and 5% CO_2_. Absorbance of the formazan complex formed through this assay was measured at 490 nm [35]. As described above, positive controls for both calcein-AM staining and the MTS assay were provided by MC3T3-E1 cells seeded on polystyrene tissue culture-treated plates, and negative control parameters were provided by Fibro-Gide^®^ wafers and polystyrene tissue culture-treated plates without any cell seeding. Negative controls were also utilized in data interpretation to account for background signal caused by Fibro-Gide^®^ wafers.

Cell proliferation and ingrowth on Fibro-Gide^®^ wafers on days 3, 5 and 7 was also assessed via histology with hematoxylin and eosin (H&E) staining.

### 2.5. In Vivo Evaluation

#### 2.5.1. Rodent Model

Female Sprague–Dawley rats (n = 12) ranging from 190–210 g were utilized. Animals were housed, cared for, and handled under standard conditions and in accordance with the Institutional Animal Care and Use Committee (IACUC) guidelines for the duration of the study. The model utilized in this experiment is a variation of a previously described critical size mandibular defect model [36,37,38] and was selected to facilitate creation of a sufficiently large defect to accommodate scaffold implantation and evaluation of tissue–scaffold integration.

#### 2.5.2. Surgical Preparation and Surgical Procedure

Rats were anesthetized with isoflurane and received a pre-operative dose of buprenorphine (0.05 mg/kg) subcutaneously (SQ). Surgical preparation included shaving fur from the left lateral neck to the left ear pinna. Aseptic skin preparation was accomplished with chlorhexidine and isopropyl alcohol. Eyes were lubricated with sterile lubricant, and rats were maintained on isoflurane inhalant anesthetic via nose cone throughout the surgical procedure.

During surgery, skin and muscle layers were sharply dissected to expose the left mandible, and a critical-sized (5 mm) circular defect was created in the ramus of the mandible utilizing a handheld microdrill (Ideal Microdrill, CellPoint Scientific, Inc. Gaithersburg, MD. USA) with 5 mm circular bit. Defects were filled with sterile collagen matrix cylinder (Fibro-Gide^®^) in either the native form or were impregnated with 10 mg (100 μL) of gentamicin (100 mg/mL; approximately 40 mg/kg dose of gentamicin). Native or impregnated collagen matrix relegated animals into either control (n = 6) or experimental group (n = 6), respectively.

No post-operative antibiotics were provided systemically or parenterally. Following surgery, animals were housed individually, provided free choice water, and maintained on a soft gel diet to minimize mechanical trauma from gnawing or chewing. These conditions were maintained for the remainder of the study. Animals were monitored every 12 h for the first five days following surgery, and additional doses of 0.05 mg/kg buprenorphine were administered SQ every 12 h for the first three days post-operatively. Four weeks post-operatively, rats were humanely euthanized via anesthetic (isoflurane) overdose and thoracotomy for sample collection.

#### 2.5.3. Computed Tomography Analysis

After sacrifice, animals were scanned using computed tomography (CT) to evaluate the defect sites. Animals were positioned in sternal recumbency on the CT table, and scanning parameters were limited to the skull of each animal. Sectional scans and 3D renderings of the regions of interest (ROIs) were collected and reviewed by a board-certified radiologist. Qualitative analysis of the defect area and surrounding bone was compiled.

#### 2.5.4. Histological Analysis

Following CT scanning, left hemimandibles were harvested, formalin fixed, and decalcified in Formical-2000 for 48 h until they could be sharply dissected without bony resistance. After decalcification, hemimandibles were transferred to 10% neutral buffered formalin and were submitted for histology (University of Tennessee College of Veterinary Medicine, Veterinary Diagnostic Laboratory, Histopathology Service). Hemimandibles were embedded within paraffin, and 4 μm decalcified sections were obtained and stained with H&E. Slides were assessed qualitatively and semi-quantitatively by investigators as well as a board-certified veterinary pathologist who was blinded to treatment groups. Evaluation parameters of the tissue–scaffold interface were based on a modified ordinal grading scale [39,40] to evaluate degree of tissue–scaffold integration and severity of inflammatory response. Tissue–scaffold integration was evaluated based on angiogenesis throughout the scaffold, connective tissue infiltration into the scaffold, cellular and connective tissue infiltration into the surrounding tissues, and fibrous tissue encapsulation. The aforementioned categories were graded based on a point system from 0 to 3 with the following categories: (0) absent, (1) mild, (2) moderate, and (3) marked. Tissue–scaffold integration had a maximum positive score of 9 points ((angiogenesis + connective tissue infiltration into scaffold + cellular and connective tissue infiltration to surrounding tissues)—fibrous tissue encapsulation). Degree of inflammatory response was evaluated based on inflammatory reaction, degree of fibrous tissue encapsulation, suppurative vs. non-suppurative cellular response and presence or absence of necrotic material. Inflammatory reaction and degree of fibrous tissue encapsulation were graded based on a point system from 0 to 3 with the following categories: (0) absent, (1) mild, (2) moderate, and (3) marked. Suppurative cellular response and presence of necrotic material were reported as nominal data, either (0) absent or (1) present. Degree of inflammatory response had a maximum severity score of 8 points (inflammatory response + fibrous tissue encapsulation + suppurative cellular response + necrosis).

### 2.6. Statistical Analysis

The effects of treatment, dose and time on response variable total gentamicin were examined using mixed model analysis for repeated measures. Ranked transformation was applied when diagnostic analysis on residuals exhibited violation of normality and equal variance assumptions using Shapiro–Wilk test and Levene’s test. Post hoc multiple comparisons were performed with Tukey’s adjustment. Statistical significance was identified as *p* values (alpha-error) at <0.05. Analyses were conducted in SAS 9.4 TS1M4 (SAS institute Inc., Cary, NC, USA). Effects of treatment, concentration and time on MTS were analyzed using repeated-measures ANOVA, with treatment and concentration as the between-subject effects with time as the repeated factor. Diagnostic analysis was conducted to exam model assumptions. Ranked transformation was applied if diagnostic analysis exhibited violation of normality and equal variance assumptions. Post hoc multiple comparisons were performed with Tukey’s adjustment. Statistical significance was identified at the level of 0.05. Analyses were conducted in SAS 9.4 TS1M7 for Windows 64× (SAS institute Inc., Cary, NC, USA). A Student’s *t* test (two-tailed, assuming homoscedasticity) was performed to evaluate for any significant differences between histological scores of control and experimental groups.

## 3. Results

### 3.1. Hydrophilicity

The 5 mm diameter biopsy punch effectively cut the Fibro-Gide^®^ material into cylinders (n = 6) with an average height and width of 5.53 ± 0.22 and 5.22 ± 0.12 mm, respectively. Cylinders could load 100 μL of PBS or gentamicin (100 or 50 mg/mL) without leaving excess residue on loading platform. This was considered to be the maximum saturation and maximum loading dose for this material size and type. Initial average dry weight of Fibro-Gide^®^ cylinders was 9.84 ± 0.57 mg. The addition of 100 μL of 50 or 100 mg/mL gentamicin resulted in a 1211.20 ± 346.11% increase in weight of material, with an average post-loading weight of Fibro-Gide^®^ cylinders equal to 128.96 ± 2.54 mg.

### 3.2. Gentamicin Elution

**Figure 1:**
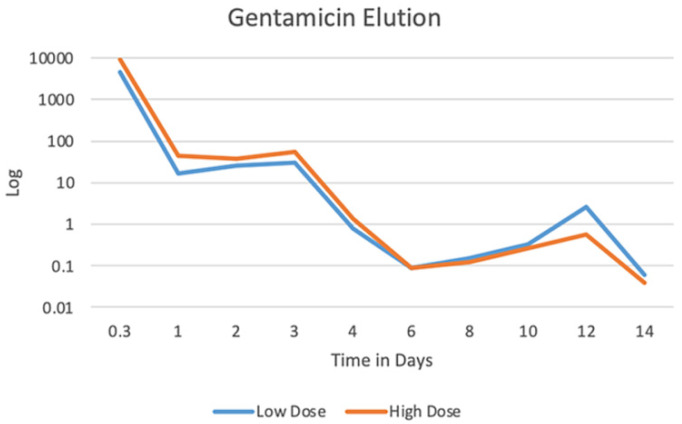
Median gentamicin elution from Fibro-Gide^®^ cylinders in PBS over a 14-day period. Log transformation applied to best visualize elution curve, including both the initial burst release and sustained lower-level release of gentamicin.

Fibro-Gide^®^ samples loaded with 5 mg gentamicin (n = 3) eluted an average of 4.95 ± 0.57 mg gentamicin throughout the 14-day period with peak elution of 4.47 ± 0.31 mg at three hours. This is equivalent to eluting 89.4 ± 6.2% of the loaded gentamicin within the first three hours. Fibro-Gide^®^ samples loaded with 10 mg gentamicin (n = 3) eluted an average of 9.97 ± 1.5 mg throughout the 14-day period with peak elution of 9.14 ± 0.36 mg at three hours. This is equivalent to eluting 91.4 ± 3.6% of the loaded gentamicin within the first three hours. Elution curve (Figure 1) demonstrates an initial burst release followed by a gradual lower-level release over the 14-day study period.

### 3.3. In Vitro Cytocompatibility

**Figure 2:**
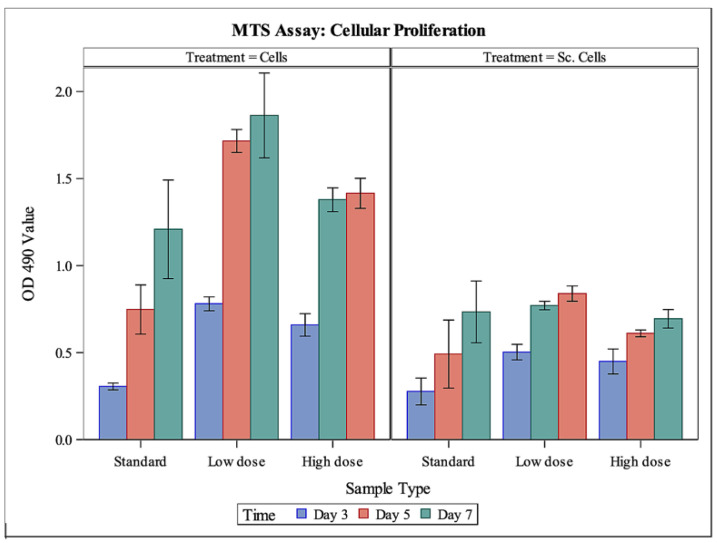
MTS assay to measure cellular proliferation. Left-side panel (cells) demonstrates mean cellular proliferation, measured through absorbance, of MC3T3-E1 cells in cell culture exposed to standard media, or a low or high dose of gentamicin, through time. Right-side panel (Sc. Cells) demonstrates mean cellular proliferation, measured through absorbance, of MC3T3-E1 cells on Fibro-Gide^®^ wafers exposed to standard media, or a low or high dose of gentamicin, through time. Cells in cell culture had a significantly higher proliferation than cells on collagen matrix (*p* < 0.0001). Sc. Cells, cells on Fibro-Gide^®^ wafers.

MTS assay results (Figure 2) showed that MC3T3-E1 cells were able to proliferate on Fibro-Gide^®^ wafers when exposed to standard αMEM growth media as well as αMEM growth media containing a low (20 μg/mL) or high (200 μg/mL) dose of gentamicin. As expected, proliferation of cells in standard cell culture treated-plates was significantly greater than proliferation of cells on Fibro-Gide^®^ wafers (*p* < 0.0001). For cells in cell culture and on scaffolds, proliferation of cells in the low- and high-dose gentamicin media was significantly greater than proliferation of cells in the standard cell culture media (*p* = 0.0002 and *p* = 0.0175, respectively). Cells in low-dose gentamicin media outperformed cells in high-dose gentamicin media (*p* = 0.0275). There were differences seen through time, with day 3 having significantly less cellular proliferation regardless of substrate than days 5 or 7 (*p* < 0.0001 and *p* < 0.0001, respectively). There were no significant differences between cellular proliferation between days 5 and 7 (*p* = 0.1985).

**Figure 3:**
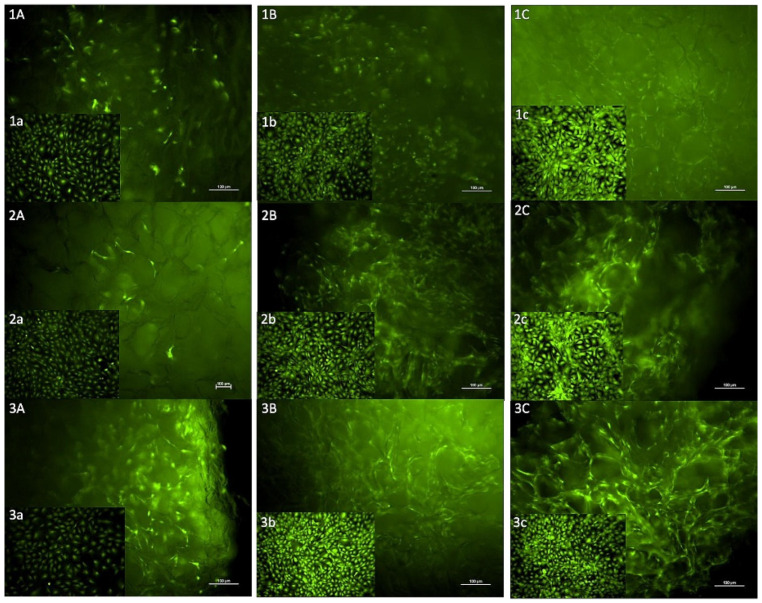
Images of calcein-AM staining of MC3T3-E1 cells on Fibro-Gide^®^ wafers with inset positive control images of MC3T3-E1 cells in cell culture. (**1A**–**1C**) Cells on matrix on days 3 (**A**), 5 (**B**), 7 (**C**) in standard media. (**1a**–**1c**) Cells in culture on days 3 (**a**), 5 (**b**), 7 (**c**), standard media. (**2A**–**2C**) Cells on matrix on days 3 (**A**), 5 (**B**), 7 (**C**) exposed to low dose gentamicin. (**2a**–**2c**) Cells in culture on days 3 (**a**), 5 (**b**), 7 (**c**), low-dose gentamicin. (**3A**–**3C**) Cells on matrix on days 3 (**A**), 5 (**B**), 7 (**C**), exposed to high-dose gentamicin. (**3a**–**3c**): Cells in culture on days 3 (**a**), 5 (**b**), 7 (**c**), high-dose gentamicin.

Calcein-AM staining showed that MC3T3-E1 cells were able to proliferate and remain viable on Fibro-Gide^®^ wafers when exposed to standard αMEM growth media as well as αMEM growth media containing a low or high dose of gentamicin. Positive controls as well as experimental samples all demonstrated an increase in numbers of fluorescent cells between days 3, 5 and 7. Images of fluorescently tagged cells highlight the three-dimensional nature and porous texture of the Fibro-Gide^®^ wafers (Figure 3).

**Figure 4:**
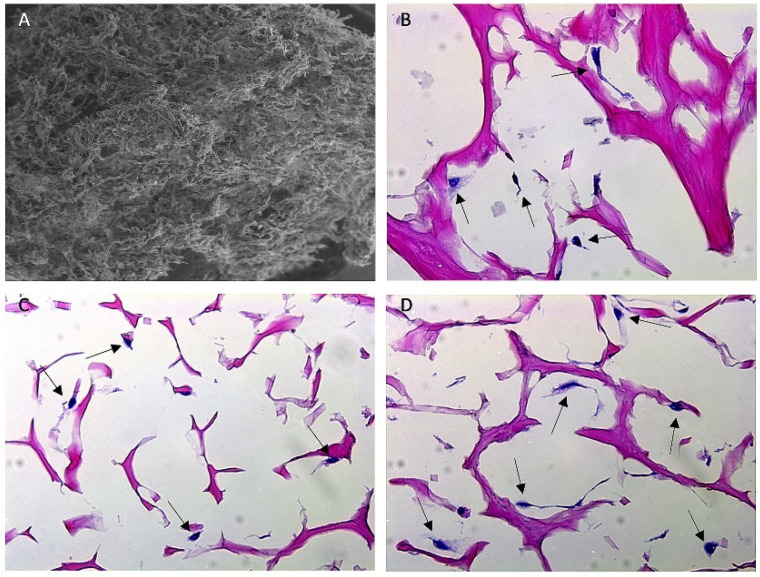
SEM and H&E-stained histology images of Fibro-Gide^®^ collagen matrix. (**A**) SEM image of Fibro-Gide^®^ cylinder. (**B**–**D**) Histology images taken at 40× magnification of Fibro-Gide^®^ seeded with MC3T3-E1 cells on days 3 (**B**), 5 (**C**), 7 (**D**) of cell culture. Dark pink material is collagen matrix, dark purple material is cell nuclei, denoted by black arrows.

Histopathology with H&E staining (Figure 4) demonstrated the porous nature of the collagen matrix and showed individual cells in various orientations throughout the matrix, confirming the hypothesis that cells can proliferate on Fibro-Gide^®^ and grow into the porous channels.

### 3.4. In Vivo Biocompatibility

#### 3.4.1. Animal Observation and Care

No anesthetic complications occurred. Animals subjectively recovered well and appeared comfortable. Animals did not develop significant facial swelling or discomfort that impeded their ability to eat, drink or interact with their environment. Objectively, incisions healed without any surgical site infections, and animals gained weight throughout the study. As a result of eating a soft-gel diet instead of a regular rodent chow pellet, one rat developed a malocclusion, or overgrowth of the incisor teeth, which resulted in secondary side effects and euthanasia prior to the intended endpoint. This animal was not included in data analysis, resulting in n = 5 control animals and n = 6 experimental animals. Otherwise, all remaining rats completed the study uneventfully, but most required corrective tooth trimming once weekly to prevent additional instances of dental malocclusion.

#### 3.4.2. CT Analysis

**Figure 5:**
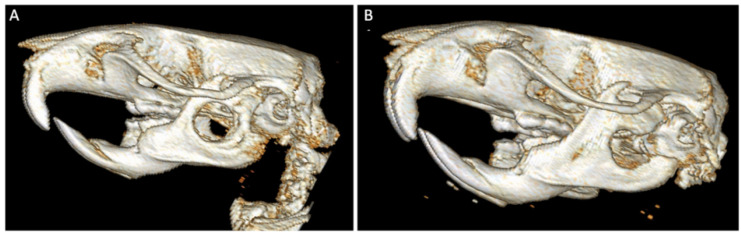
Three-dimensional CT scan renderings of mandibular defect. Image (**A**) displays the ideal placement of the critical-sized (5 mm diameter), full-thickness bone defect on rodent hemimandible. Image (**B**) demonstrates defect placement that is too far caudal on the hemimandible. Both images provide appreciation for the difficulty of completing quantitative analysis due to the small defect and animal size.

One month after surgery, qualitative and semi-quantitative CT analysis of the control group demonstrated mild to moderate periosteal reaction, absent to mild medullary sclerosis, absent to mild soft tissue swelling, and no evidence of bone lysis or bone healing. CT analysis of the experimental group demonstrated mild to moderate periosteal reaction, mild medullary sclerosis, absent to mild soft tissue swelling, and no evidence of bone lysis or healing. Qualitative CT analysis also demonstrated variation in defect placement (Figure 5), including defect placement that extended off the caudal border of the mandible and placement that extended into the oral cavity.

#### 3.4.3. Histological Analysis

Control group: Control animals (those that received a defect and non-impregnated collagen matrix) had an average tissue–scaffold integration score of 4.2 ± 2.77 (maximum score of 9) and an average inflammatory score of 4.8 ± 1.92 (maximum score of 8, denoting most inflammatory processes). All but one of these sections demonstrated similar qualities and degrees of angiogenesis, fibrous tissue encapsulation, connective tissue infiltration into the collagen matrix, and mononuclear cellular population. One specimen (rat #9) differed greatly from the other control samples, with a tissue–scaffold integration score of 0 and a degree of inflammation score of 8. This animal displayed severe, suppurative inflammation with degenerative neutrophils and areas of necrosis within the collagen matrix. On CT analysis, this animal also suffered a pathologic fracture ± osteomyelitis (bone infection). One animal (rat #11) had the presence of few multi-nucleated giant cells (MNGCs) distributed within the periphery of the collagen matrix. This animal had no surgical complications or abnormalities on CT analysis.

Treatment group: Experimental animals (those that received a defect and collagen matrix impregnated with 40 mg/kg gentamicin) had an average tissue–scaffold integration score of 4.0 ± 2.0 (maximum score of 9) and an average inflammatory score of 5.5 ± 2.16 (maximum score of 8, denoting most inflammatory process). Similar to control specimens, these sections demonstrated similar qualities, extent of angiogenesis, fibrous tissue encapsulation, connective tissue infiltration into the collagen matrix, and mononuclear cellular population. Two specimens (rats #2, 10) differed greatly from other experimental samples, with tissue–scaffold integration scores of 1 and 2, respectively, and degree of inflammation scores of 8. These animals displayed severe, suppurative inflammation with degenerative neutrophils and areas of necrosis within the collagen matrix. Neither of these animals had evidence of osteomyelitis on CT analysis, and neither animal suffered a pathologic surgical complication. Three animals (rats #8, 10 and 12) had one to few MNGCs present around the periphery of the collagen matrix. Two rats (#8 and 12) had some extension of the surgical defect into the caudal mandibular border.

**Figure 6:**
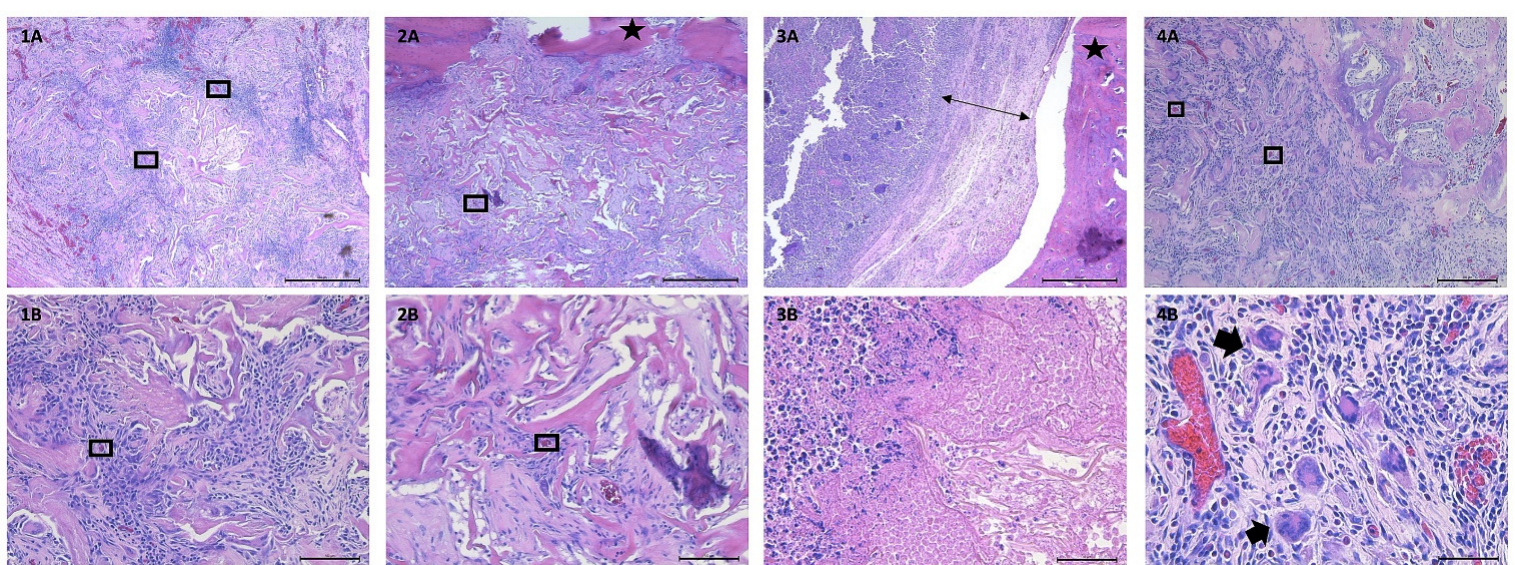
Histology images from control and experimental animals. (**1A**) 5× magnification, demonstrating cellular and connective tissue infiltration into the native collagen matrix (control animal, rat #7). (**1B**) 20× magnification, demonstrating cellular and connective tissue infiltration into native collagen matrix (control animal, rat #7). (**2A**) 5× magnification, demonstrating cellular and connective tissue infiltration into antibiotic-loaded collagen matrix (experimental animal, rat #4). (**2B**) 20× magnification, demonstrating cellular and connective tissue infiltration into antibiotic-loaded collagen matrix (experimental animal, rat #4). (**3A**) 5× magnification, demonstrating thick ring of fibrous tissue separating bone from native collagen matrix and surrounding suppurative inflammation (control animal, rat #9). (**3B**) 40× magnification, demonstrating dense population of neutrophils (left side of image), and necrotic cellular infiltration into native collagen matrix (control animal, rat #9). (**4A**) 10× magnification, demonstrating cellular and connective tissue infiltration into antibiotic-loaded collagen matrix (experimental animal, rat #12). (**4B**) 40× magnification, demonstrating presence of multinucleated giant cells, as well as blood vessels, throughout antibiotic-loaded collagen matrix (experimental animal, rat #12). Boxes are surrounding select blood vessels to highlight angiogenesis throughout the collagen matrix. Stars denote bone. Double-sided arrow highlights the thick rim of fibrous connective tissue. Thick arrows point at select multi-nucleated giant cells.

Between both groups, all specimens displayed mononuclear cellular infiltrate into the collagen matrix. Specimens varied in quantity of fibrous tissue surrounding the collagen matrix as well as infiltration of blood vessels into the center of the collagen matrix. Four specimens (36% of animals) displayed evidence of MNGCs (one control specimen, three experimental specimens). Three specimens (27% of animals) displayed severe, degenerative, suppurative inflammation (one control specimen, two experimental specimens). There were no statistically significant differences detected between tissue–scaffold integration scores of control and experimental animals (*p* = 0.89) or between degrees of inflammation between the two groups (*p* = 0.58). Representative images of the described findings are displayed in Figure 6.

## 4. Discussion

The results from this work indicate that commercially available collagen matrix Fibro-Gide^®^ can support the proliferation and viability of MC3T3-E1 cells in vitro, even in the presence of gentamicin. Gentamicin is an aminoglycoside antibiotic that is known to possess an acidic pH, which can alter local in vitro or tissue environments and that can alter mitochondrial respiration, enhancing the generation of reactive oxygen species (ROS), such as hydrogen peroxide and superoxide [41]. ROS are recognized to cause DNA damage, which can lead to cell death. ROS also can cause decreased cellular proliferation and decreased angiogenesis and can stimulate inflammation [42]. Therefore, the ability of cells to proliferate in the presence of gentamicin in vitro is important. Histology of the cell-loaded collagen matrix demonstrated the ability of MC3T3-E1 cells to proliferate and grow within the porous channel system provided by Fibro-Gide^®^. This finding supports the ability of the collagen matrix to support tissue ingrowth, which supports its biocompatibility and subsequent biodegradation. On the basis of these studies, Fibro-Gide^®^ can be loaded with and elute the antimicrobial gentamicin. In vitro elution was characterized by an initial burst release followed by sustained lesser release for the 14-day study period. The described release kinetics fall into a pattern typical of devices loaded by impregnation rather than specific molecule-linkages or stimuli-responsive systems [9,20]. Impregnation via material soaking in antibiotic solutions is the most common method used for loading of collagen materials [20].

These in vitro data support an in vivo investigation to determine biocompatibility of the material when impregnated with a high dose of antimicrobial. In vivo investigation in a rat mandible defect indicated satisfactory tissue–scaffold integration and mild degrees of inflammation given the clinical scenario of the critical-sized mandibular defect, which inherently induces tissue trauma and associated inflammation. Histology demonstrated a mixture of connective tissues infiltrating throughout the collagen matrix along with the presence of mononuclear cellular infiltrate. Mononuclear cells, such as lymphocytes, are components of the host immune system and play important, multifaceted roles in inflammation and tissue healing [24]. Lymphocytes have been recognized to not only respond to acute inflammation and to be associated with chronic inflammation, but also to regulate angiogenesis, tissue healing and regeneration [43,44]. Finding formed connective tissue throughout the collagen matrix instead of solely fibrous connective tissue surrounding the collagen matrix supports the in vivo biocompatibility of the device [45,46], even while loaded with gentamicin. The presence of MNGCs is controversial [47,48]. When there is a significant or overwhelming population of MNGCs in conjunction with a fibrotic ring of tissue and/or dense neutrophilic inflammation surrounding an implanted device, it can be deduced that the body is mounting a foreign body response to that material [45]. However, there are also situations in which material degradation can induce the formation of a smaller number of MNGCs [23,49]. Recognizing that Fibro-Gide^®^ is a resorbable collagen matrix, it is reasonable to believe that the limited number of MNGCs noted along the periphery of four scaffolds is reflective of the intended degradation process and that it is not an adverse foreign body response.

While the majority of specimens indicated satisfactory tissue–scaffold integration and degrees of inflammation, three specimens demonstrated marked suppurative inflammation and areas of necrosis throughout the collagen matrix. One of these specimens was associated with a pathologic fracture and evidence of osteomyelitis on CT scan. It is likely that the pathologic fracture occurred peri-operatively and resulted in a local inflammatory environment that encouraged the development of a suppurative response. This may have been exacerbated by mobility of the implant within the defect site due to the pathologic fracture or exposure to the oral cavity, which can rapidly increase the degradation rate of collagen [50,51]. In the other two specimens, the animals had an unsatisfactory tissue–scaffold reaction. Suppurative tissue–scaffold reactions in these two experimental specimens may be due to individual immune response, mobility of the implant within the defect site, bacterial contamination, or exposure to the oral cavity, which can increase the degradation rate of collagen [50,51]. Gentamicin, although it carries many benefits, also has the risk of altering local tissue environments by promoting an acidic pH and by enhancing the generation of ROS [41]. It is possible that the enhanced presence of ROS by action of gentamicin may have stimulated an increased rate of apoptosis, leading to the necrotic foci throughout the collagen matrix and stimulating the intense inflammatory tissue response. It is reasonable to consider the possibility that other antibiotics may impact the tissue–scaffold interface and degrees of inflammation differently. Another consideration when interpreting in vivo biocompatibility data is the variation in defect placement and the potential contribution of variation ± pathologic fractures on the tissue–scaffold interface visualized on histopathology.

SSIs can be superficial or deep and may involve medical implants or areas of tissue loss, whether that tissue loss is due to trauma, is resultant from surgery, or is secondary to revision procedures such as debridement. When dealing with SSIs that extend to deeper tissue planes, a locally implantable drug delivery device may add strength to the treatment regimen. Similarly, when SSIs involve any aspect of tissue loss or destruction, the ability to treat infection while aiding in tissue regeneration is likely to restore form and function to the patient more rapidly and with fewer interventions than traditional treatment strategies. Additionally, the potential to utilize an FDA-approved tissue regeneration product as a dual-platform device for drug delivery holds exceptional opportunities to prevent bacterial infection in local tissue environments. The strength in this transition comes not only in the consistency and availability of the product, but also in healthcare provider familiarity with the product. When considering consistency and availability, a major advantage of utilizing a currently available material is the production process. Many investigations into biomaterials for drug delivery and tissue regeneration demonstrate positive results when the materials are fabricated in small batches, but they encounter significant technical and performance challenges once scaling up material production is attempted. Therefore, the investigation of a commercially available collagen matrix that has previously demonstrated appropriate biocompatibility [23,24,46] for utilization as a dual-platform device is a reasonable undertaking and provides valuable information.

## 5. Limitations

Limitations within this work include small sample size, variability in defect placement and lack of concurrent in vivo drug elution kinetic characteristics. Greater consistency in defect placement in a larger number of animals is ideal to maintain a uniform population and would remove the consideration of defect placement from histological analysis. Another limitation of this study was our imaging modality. While CT provided useful information and valuable 3D renderings of mandibular defects, microCT would have provided finer detail and allowed for quantitative analysis of bone defects, rather than solely qualitative and semi-quantitative measurements. There is always variation in histologic appearance based on inter-animal variation, exact specimen positioning within the paraffin block, and in particular, the 4 μm sections obtained.

## 6. Conclusions and Future Directions

This work was performed to assess the overall fitness of commercially available collagen matrix Fibro-Gide^®^ for utilization as a locally implantable drug delivery device, in conjunction with the product-labeled usage of a soft tissue regeneration device. To assess overall fitness, we began with the following three hypotheses: the device (1) would be able to load and elute antimicrobials, (2) would be cytocompatible in vitro, and lastly, (3) a high dose of antimicrobials loaded within the device would negatively impact the tissue–scaffold interface in vivo. We demonstrated that Fibro-Gide^®^ is able to load and elute the antimicrobial gentamicin, with the expected elution kinetics, confirming the first hypothesis. We demonstrated cytocompatibility in vitro with or without the presence of gentamicin, confirming the second hypothesis. Lastly, we found no significant differences between the tissue–scaffold interface of animals that received either the native device or device loaded with a high dose (40 mg/kg) of the antimicrobial gentamicin, therefore rejecting the third hypothesis. Based on these parameters, we conclude that this commercially available collagen matrix holds value for further consideration to be utilized as a local drug delivery device, especially in situations where soft-tissue regeneration or augmentation is desired.

To further investigate the value of this collagen matrix for use as a dual-platform device, a reasonable initial step is to perform a similar experiment utilizing a larger number of animals and a slightly smaller defect size. This approach would likely result in more uniform defect placement and would more clearly elucidate the impact of a high dose of gentamicin on the tissue–scaffold interface, eliminating the concern of pathologic fractures leading to suppurative tissue responses. Further investigations may focus on a wider array of drugs to evaluate drug elution characteristics and subsequent device compatibility, or may focus on the fine tuning of drug elution kinetics, either by assessing in vivo elution [20] or by alternative drug-loading strategies. Situations of particular value for utilization of this collagen matrix as a dual-platform device include surgical site infections that involve soft tissue loss and cases where systemic antimicrobial usage needs to be minimized.

## Data Availability

The data presented in this study are available on request from the corresponding author.
